# Supplementary material for: Detection of Mosaic Absence of Heterozygosity (AOH) Using Low-Pass Whole Genome Sequencing in Prenatal Diagnosis: A Preliminary Report
Source: Diagnostics (Basel). 2023 Sep 9;13(18):2895. doi: 10.3390/diagnostics13182895 (PMC10529865; doi:10.3390/diagnostics13182895)
Supplement: Supplementary file 1 [file diagnostics-13-02895-s001.zip › Supplementary figure.pptx]

## Slide 1
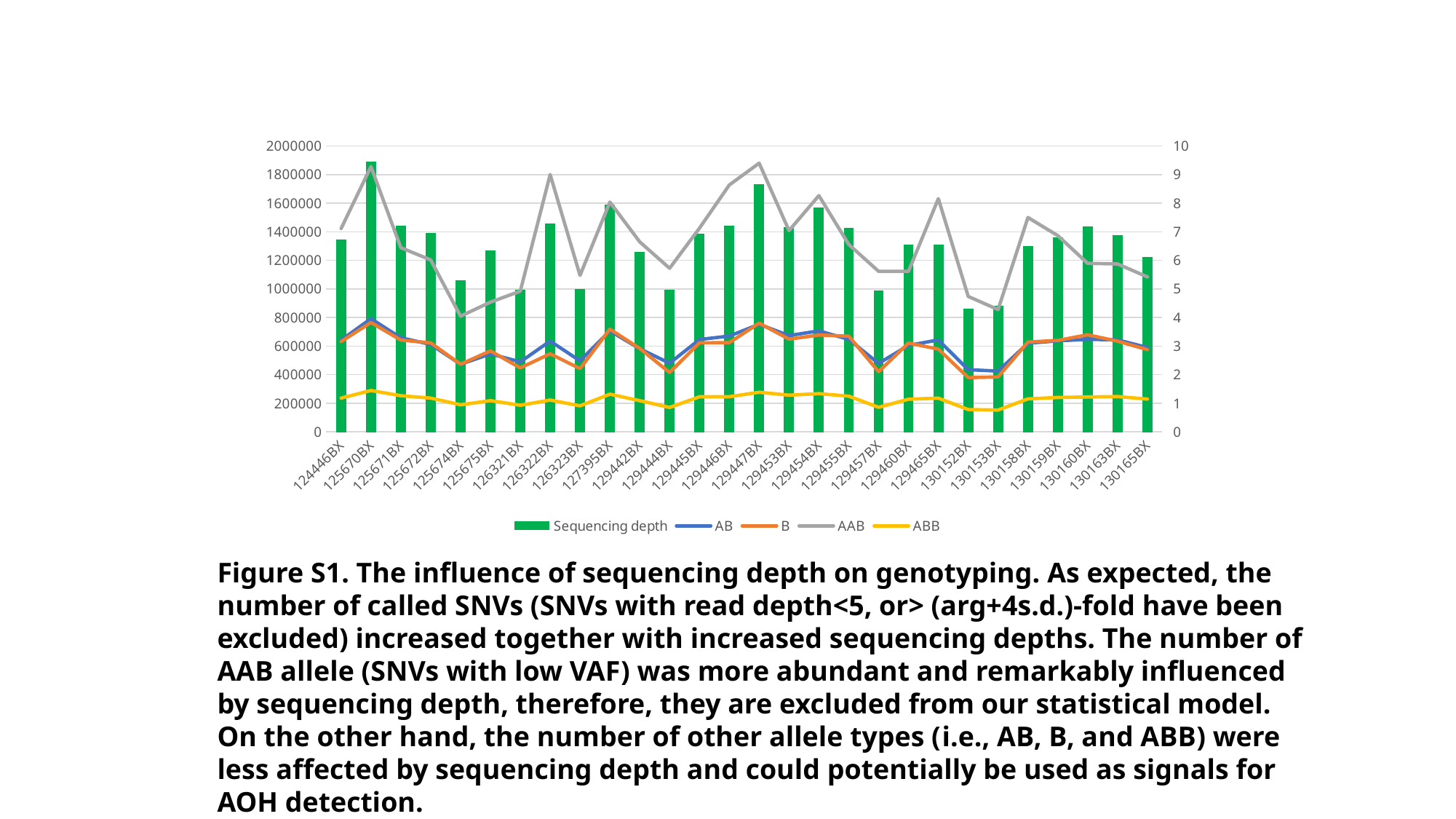

### Chart
| Category | Sequencing depth | AB | B | AAB | ABB |
|---|---|---|---|---|---|
| 124446BX | 6.72 | 641320.0 | 631529.0 | 1422672.0 | 236073.0 |
| 125670BX | 9.45 | 792257.0 | 764970.0 | 1856007.0 | 289263.0 |
| 125671BX | 7.2 | 657909.0 | 642079.0 | 1288628.0 | 252400.0 |
| 125672BX | 6.95 | 611578.0 | 622636.0 | 1202928.0 | 235881.0 |
| 125674BX | 5.28 | 472796.0 | 472723.0 | 809155.0 | 189035.0 |
| 125675BX | 6.34 | 545142.0 | 566366.0 | 907691.0 | 218219.0 |
| 126321BX | 4.95 | 489653.0 | 447233.0 | 984260.0 | 186191.0 |
| 126322BX | 7.27 | 634950.0 | 545949.0 | 1800588.0 | 222303.0 |
| 126323BX | 4.98 | 496771.0 | 442070.0 | 1094807.0 | 181942.0 |
| 127395BX | 7.95 | 706413.0 | 718741.0 | 1608007.0 | 263235.0 |
| 129442BX | 6.29 | 581303.0 | 583184.0 | 1329432.0 | 218050.0 |
| 129444BX | 4.95 | 480369.0 | 415106.0 | 1144728.0 | 170393.0 |
| 129445BX | 6.93 | 646039.0 | 622834.0 | 1425357.0 | 244955.0 |
| 129446BX | 7.2 | 670110.0 | 623773.0 | 1727369.0 | 245709.0 |
| 129447BX | 8.64 | 752801.0 | 760949.0 | 1879986.0 | 277032.0 |
| 129453BX | 7.14 | 673379.0 | 648509.0 | 1408174.0 | 256564.0 |
| 129454BX | 7.84 | 706637.0 | 677309.0 | 1653165.0 | 267322.0 |
| 129455BX | 7.13 | 646929.0 | 669423.0 | 1311285.0 | 249388.0 |
| 129457BX | 4.94 | 478664.0 | 421101.0 | 1122964.0 | 171736.0 |
| 129460BX | 6.54 | 604872.0 | 621041.0 | 1122634.0 | 228129.0 |
| 129465BX | 6.53 | 643033.0 | 578908.0 | 1632045.0 | 235727.0 |
| 130152BX | 4.3 | 434672.0 | 379330.0 | 947611.0 | 156030.0 |
| 130153BX | 4.4 | 424871.0 | 383884.0 | 855710.0 | 152205.0 |
| 130158BX | 6.49 | 620199.0 | 628326.0 | 1499910.0 | 230505.0 |
| 130159BX | 6.8 | 636736.0 | 639588.0 | 1372930.0 | 240486.0 |
| 130160BX | 7.17 | 647029.0 | 679075.0 | 1179099.0 | 243829.0 |
| 130163BX | 6.88 | 643490.0 | 634246.0 | 1175002.0 | 246720.0 |
| 130165BX | 6.1 | 589932.0 | 574960.0 | 1084186.0 | 228916.0 |Figure S1. The influence of sequencing depth on genotyping. As expected, the number of called SNVs (SNVs with read depth<5, or> (arg+4s.d.)-fold have been excluded) increased together with increased sequencing depths. The number of AAB allele (SNVs with low VAF) was more abundant and remarkably influenced by sequencing depth, therefore, they are excluded from our statistical model. On the other hand, the number of other allele types (i.e., AB, B, and ABB) were less affected by sequencing depth and could potentially be used as signals for AOH detection.

## Slide 2
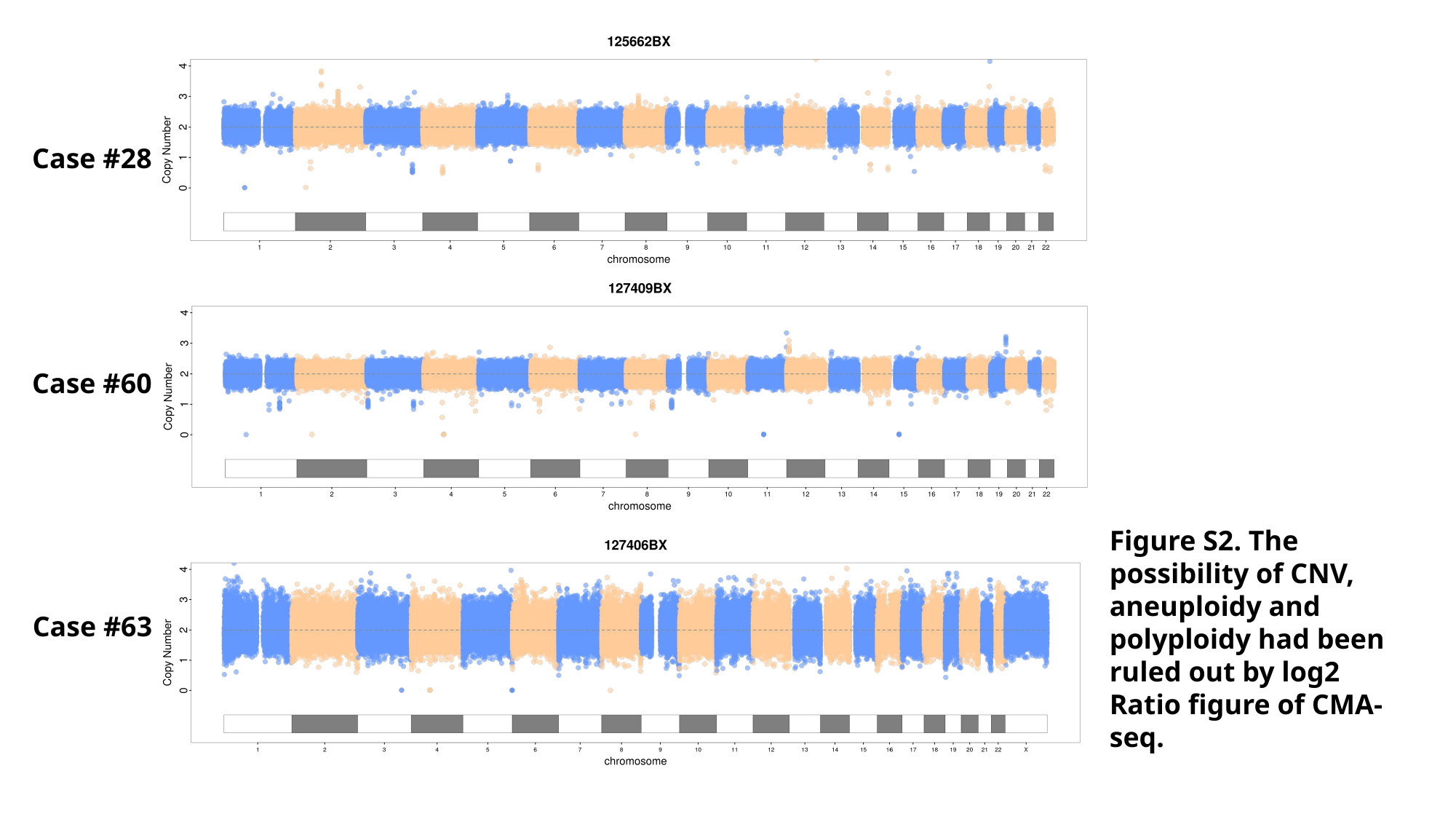

Case #28
Case #60
Case #63
Figure S2. The possibility of CNV, aneuploidy and polyploidy had been ruled out by log2 Ratio figure of CMA-seq.

## Slide 3
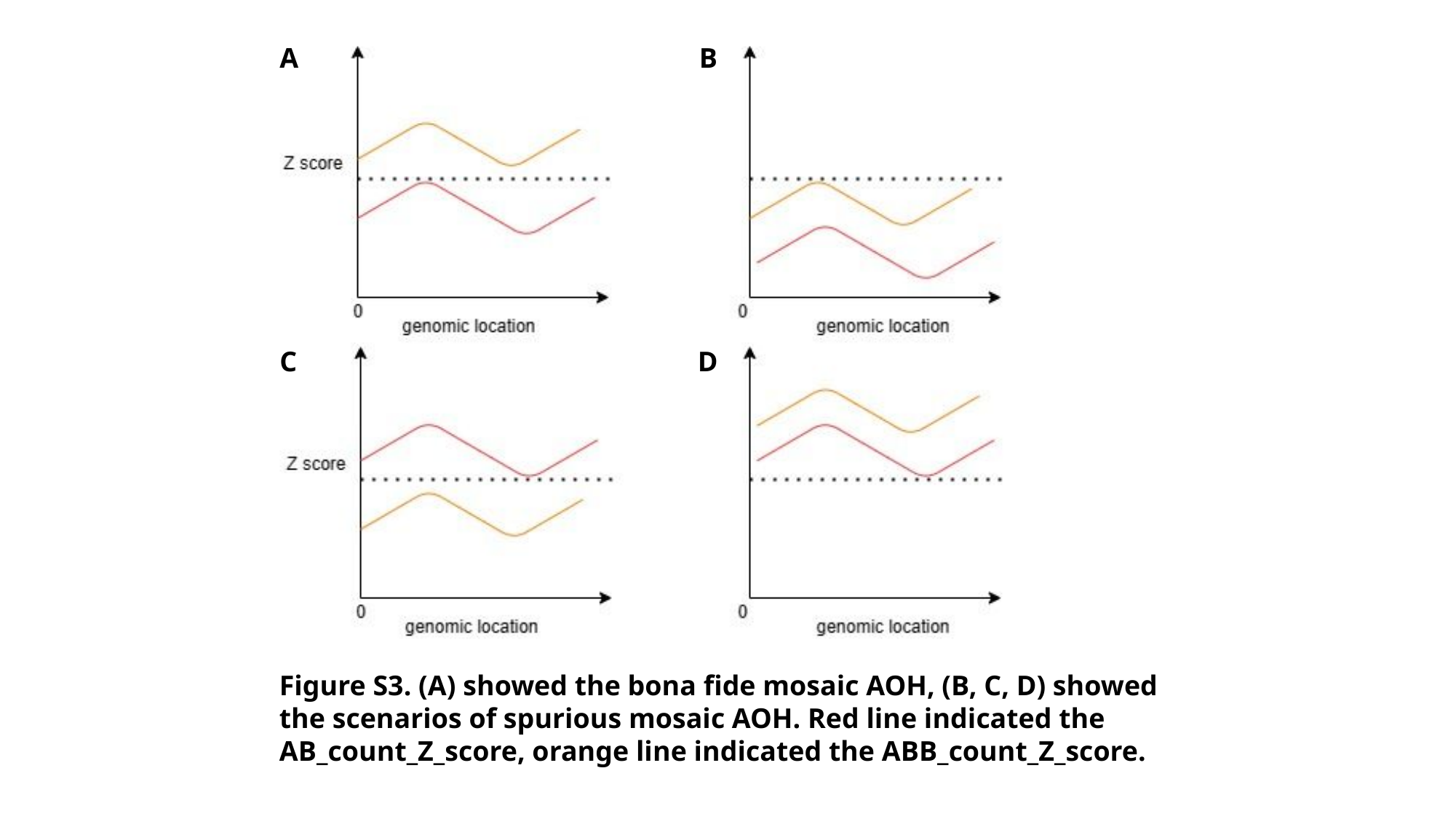

A
B
C
D
Figure S3. (A) showed the bona fide mosaic AOH, (B, C, D) showed the scenarios of spurious mosaic AOH. Red line indicated the AB_count_Z_score, orange line indicated the ABB_count_Z_score.

## Slide 4
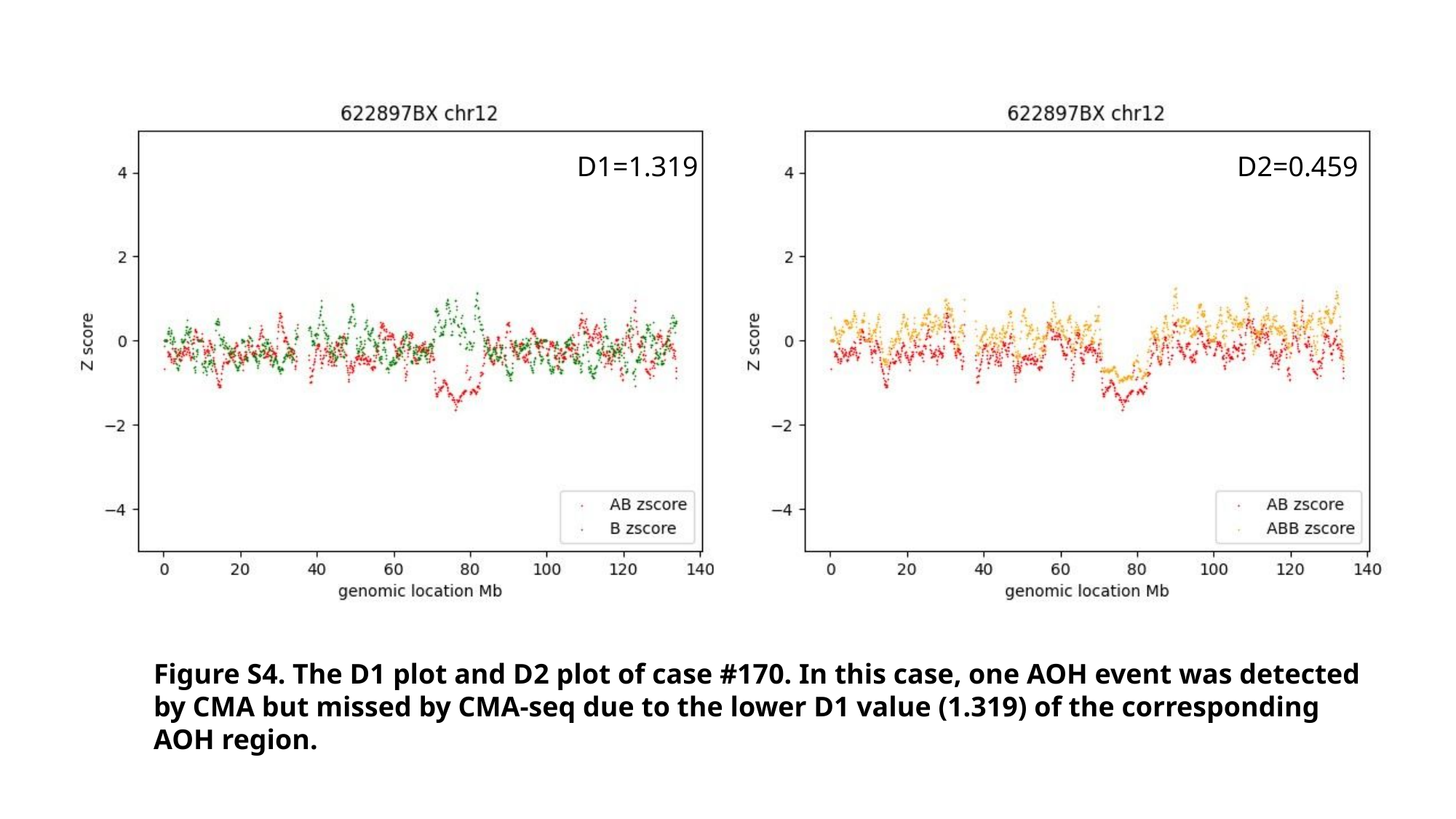

D1=1.319
D2=0.459
Figure S4. The D1 plot and D2 plot of case #170. In this case, one AOH event was detected by CMA but missed by CMA-seq due to the lower D1 value (1.319) of the corresponding AOH region.
